# Phenothiazines Enhance Mild Hypothermia-induced Neuroprotection via PI3K/Akt Regulation in Experimental Stroke

**DOI:** 10.1038/s41598-017-06752-5

**Published:** 2017-08-07

**Authors:** Hong An, Yunxia Duan, Di Wu, James Yip, Omar Elmadhoun, Joshua C. Wright, Wenjuan Shi, Kaiyin Liu, Xiaoduo He, Jingfei Shi, Fang Jiang, Xunming Ji, Yuchuan Ding

**Affiliations:** 1 China-America Institute of Neuroscience, Xuanwu Hospital, Capital Medical University, Beijing, China; 2 Department of Neurosurgery, Wayne State University School of Medicine, Detroit, MI USA; 3 Cerebrovascular Diseases Research Institute, Xuanwu hospital, Capital Medical University, Beijing, China

## Abstract

Physical hypothermia has long been considered a promising neuroprotective treatment of ischemic stroke, but the treatment’s various complications along with the impractical duration and depth of therapy significantly narrow its clinical scope. In the present study, the model of reversible right middle cerebral artery occlusion (MCAO) for 2 h was used. We combined hypothermia (33–35 °C for 1 h) with phenothiazine neuroleptics (chlorpromazine & promethazine) as additive neuroprotectants, with the aim of augmenting its efficacy while only using mild temperatures. We also investigated its therapeutic effects on the Phosphatidylinositol 3 kinase/Protein kinase B (*PI3K*/*Akt*) apoptotic pathway. The combination treatment achieved reduction in ischemic rat temperatures in the rectum, cortex and striatum significantly (*P* < 0.01) faster than hypothermia alone, accompanied by more obvious (*P* < 0.01) reduction of brain infarct volume and neurological deficits. The combination treatment remarkably (*P* < 0.05) increased expression of p-Akt and anti-apoptotic proteins (Bcl-2 and Bcl-xL), while reduced expression of pro-apoptotic proteins (AIF and Bax). Finally, the treatment’s neuroprotective effects were blocked by a p-Akt inhibitor. By combining hypothermia with phenothiazines, we significantly enhanced the neuroprotective effects of mild hypothermia. This study also sheds light on the possible molecular mechanism for these effects which involves the *PI3K*/*Akt* signaling and apoptotic pathway.

## Introduction

Stroke is a leading cause of devastating, long term disability worldwide leading to significant social and economic global burden. Despite promising outcomes in animal studies, all new neuroprotective agents have failed in clinical trials^1^. Therapeutic hypothermia (TH) is one of very few approaches that have shown encouraging neuroprotective effects in both animal and clinical studies^2–4^. TH has been extensively investigated in multiple studies to better understand its outcomes. The Copenhagen stroke study showed that a 1 °C increase in body temperature on admission independently predicts a 30% relative increase in long-term mortality risk^5^. Conversely, Burk *et al*. identified that hypothermia reduces infarct volumes by up to 90% in ischemic rodent models^6^. However, the delayed onset of brain hypothermia and various side effects associated with the impractical low temperature needed to achieve superior outcomes have limited its clinical applications^7, 8^. This warrants the development of novel approaches to enhance neuroprotection with TH while using only mild temperatures.

Phenothiazine neuroleptics have been shown to have antipsychotic and sedative effects^9, 10^. Chlorpromazine and promethazine (C + P) are two of the oldest and well-studied drugs from this class, which serve as prototypes for phenothiazines^11^. Due to their high lipophilicity, C + P easily pass the blood-brain barrier (BBB) leading to depressive and artificial hibernation-like effects on the central nervous system (CNS)^12^. Additionally, C + P promote vasodilation and inhibit shivering, thus allowing for a smooth and rapid fall in body temperature^13^. Promethazine also inhibits the permeability of mitochondria during ischemia and alleviates ischemic injury^14, 15^.

Apoptosis is a major process that occurs after brain ischemia and is modulated by a very complex cellular pathway. The molecular underpinnings of ischemia-induced apoptotic cell degradation, which is one of the major factors that contribute to the spread of neuronal cell loss, has been extensively studied^16^. However, there is still a scarcity of knowledge on how the apoptotic cascade can be modulated by various treatments, including TH^17, 18^. During ischemia, pro-apoptotic proteins, such as Caspase-3, Bax, and apoptosis-inducing factor (AIF), become up-regulated and are thought to be the major causes of neuronal injury^19^. On the other hand, Bcl-2 and Bcl-xL are examples of anti-apoptotic proteins that play a critical role in cellular survival. Bcl-2 and its relative, Bcl-xL, are oncogene-derived proteins that function as repressors of cell death^20^. A key regulator of the apoptotic process is the Phosphatidylinositol 3 kinase/Protein kinase B (*PI3K*/*Akt*) signal pathway. Phosphorylated Akt enhances cell survival via promoting anti-apoptotic factors expression (Bcl-2, Bcl-xL) and suppressing pro-apoptotic proteins (AIF, Bax)^21–23^.

We recently demonstrated dose-dependent neuroprotection induced by C + P in severe transient and permanent ischemic stroke^24^. We previously also showed a synergetic neuroprotective effect from combining mild hypothermia with low doses of C + P^25^. In the current study, we will study the mechanisms underlying phenothiazine/hypothermia-induced neuroprotection, especially on *PI3K*/*Akt* and the apoptotic signaling pathway. The result of the present study would comprehensively confirm the potential benefit of the therapy in stroke treatment, which may warrant a clinical study.

## Results

### Brain and body temperature

**Figure 1:**
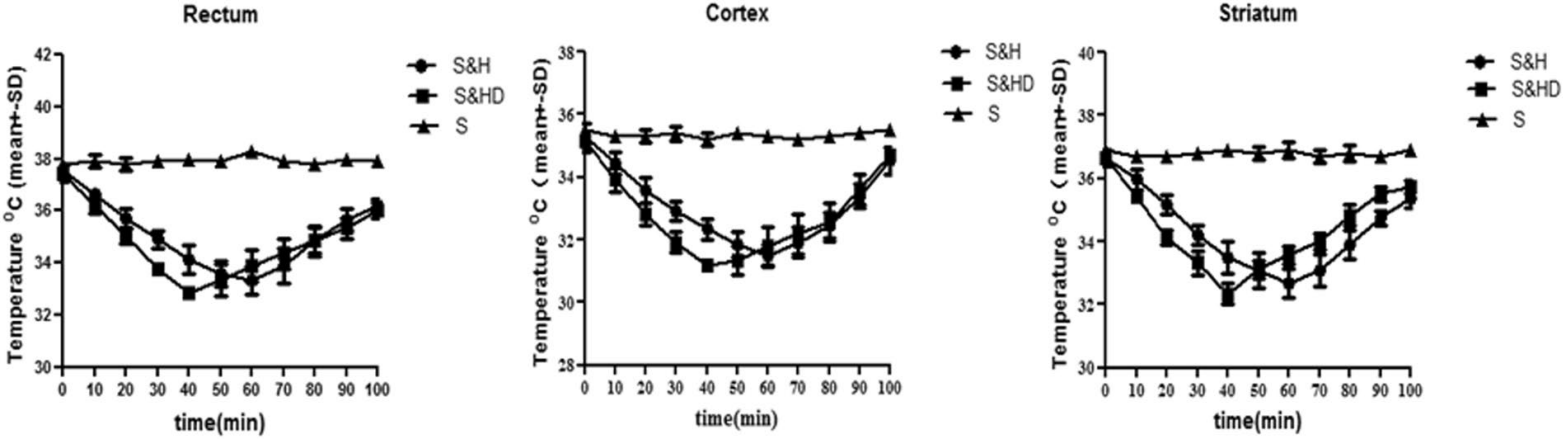
Reduction in temperature with C + P and/or mild hypothermia in the (**a**) rectum, (**b**) cortex and (**c**) striatum was measured in a time-dependent manner. As compared to stroke without treatment (S), ANOVA analyses indicated that combination therapy of C + P and mild hypothermia (S&HD) (squares) significantly induced a reduction in temperature to its maximum within 40 min and lasting up to another hour after reperfusion, whereas it took 20 min longer to get its maximum reduction in temperature by mild hypothemia alone (S&H) (circles) (**P < 0.01).

In the stroke without treatment group (S), temperatures in the rectum, cortex, and striatum were maintained within normal range (37.8–38.3 °C) with a feedback-regulated heating pad. In ischemic rats that received hypothermia and C + P (S&HD) immediately after the onset of reperfusion, temperatures in the rectum (37.42 ± 0.3 °C) (Fig. 1a), cortex (35.13 ± 0.31 °C) (Fig. 1b) and striatum (36.7 ± 0.2 °C) (Fig. 1c) were rapidly reduced to 35.0 ± 0.3 °C, 32.8 ± 0.4 °C and 34.1 ± 0.2 °C, respectively, within 20 min. Temperatures were further reduced to its lowest levels at 32.9 ± 0.1 °C in the rectum, 31.17 ± 0.2 °C in the cortex and 32.4 ± 0.3 °C in the striatum within 40 min. Temperatures were maintained below 35 °C for 1 h before gradually returning to 36.0 ± 0.3 °C in the rectum, 34.7 ± 0.3 °C in the cortex, and 35.7 ± .0.2 °C in the striatum at 100 min after reperfusion. In the stroke + hypothermia group (S&H), temperatures in the rectum (Fig. 1a), cortex (Fig. 1b) and striatum (Fig. 1c) were 37.5 ± 0.2 °C, 35.3 ± 0.4 °C, and 36.7 ± 0.2 °C respectively. Temperatures decreased gradually but took 10 min longer to reach the target temperature of 34.9 ± 0.3 °C in the rectum, 32.9 ± 0.3 °C in the cortex and 34.2 ± 0.3 °C in the striatum (*P* < 0.01). Temperatures were further reduced to its lowest levels at 33.4 ± 0.6 °C in the rectum, 31.5 ± 0.4 °C in the cortex and 32.7 ± 0.4 °C in the striatum within 60 min. Mild hypothermia was also maintained for 1 h. At 100 min of reperfusion, temperatures were gradually returned to 36.2 ± 0.3 °C in the rectum, 34.5 ± 0.4 °C in the cortex, and 35.4 ± 0.3 °C in the striatum.

### Infarct volume and neurological deficit

**Figure 2:**
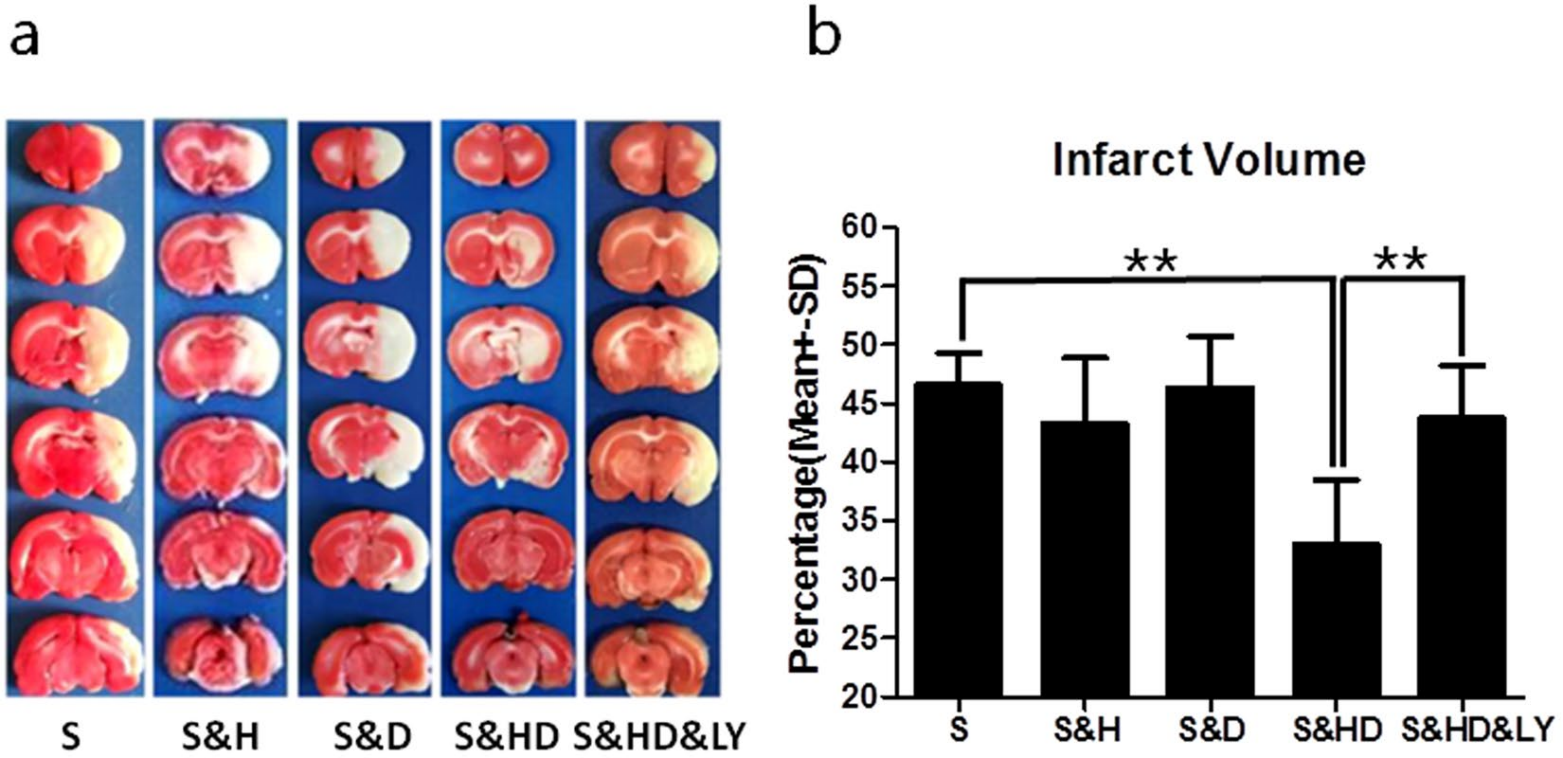
Infarct volume reduction by combination therapy of mild hypothermia and C + P. (**a**) TTC histology demonstrating infarct volume reduction in the penumbra region of ischemic territory supplied by MCA with mild hypothermia (S&H), C + P (S&D), combination therapy (S&HD), and combination therapy + p-Akt inhibitor (LY294002) (S&HD&LY). (**b**) Percentage of infarct volume reduction (Mean ± SD) with mild hypothermia (S&H), C + P (S&D), combination therapy (S&HD), and combination therapy +p-Akt inhibitor (LY294002) (S&HD&LY). Combination therapy largely and significantly reduced the infarct volume, and treatment with LY294002 reversed the combination therapy’s neuroprotective effect, implicating a role in the *PI3K*/*Akt* signaling pathway. **P < 0.01, n = 7 per group.

**Figure 3:**
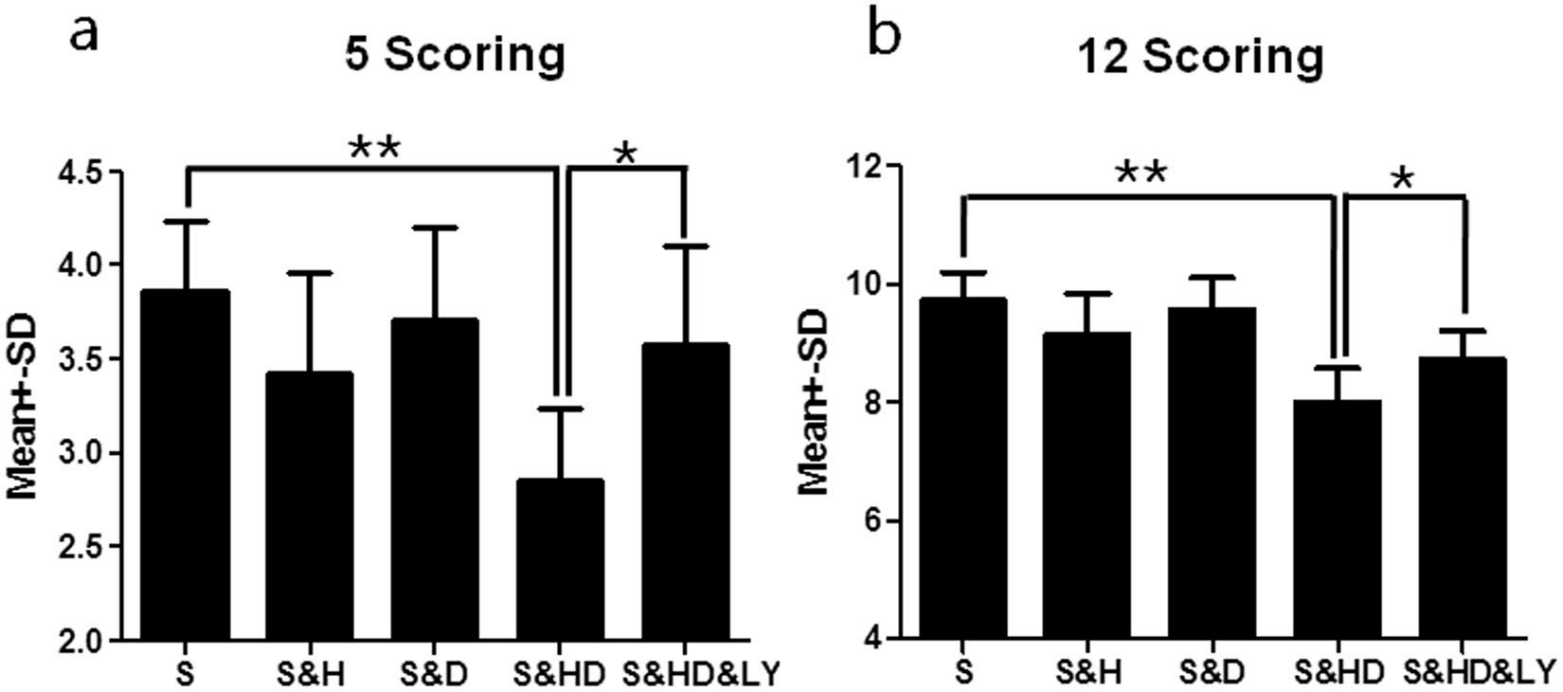
Neurological deficit (n = 7 per group) after combination therapy of mild hypothermia and C + P, using the 5-score system (**a**) and 12-score system (**b**). ANOVA analyses indicated that combination therapy (S&HD) significantly (**P < 0.01) reduced neurological deficits. Monotherapy with either mild hypothermia (S&H) or C + P (S&D) alone did not significantly improve outcomes in neuroprotection. Treatment with p-Akt inhibitor (LY294002) (S&HD&LY) reversed the combination therapy’s effect in neurological improvement (*P < 0.05).

At 24 h of reperfusion, the stroke without treatment group (S) showed an infarct volume of 45.1 ± 1.8% (Fig. 2). Mild hypothermia (S&H) slightly, but not significantly, reduced the infarct volume to 40.7 ± 3.2% (*P* = 0.262). While the use of low dose C + P (S&D) did not induce neuroprotection (47.6 ± 1.9%, *P* = 0.515), their combination with mild hypothermia (S&HD) largely and significantly reduced infarct volume to 30.7 ± 3.4% (*F*_(4,30)_ = 6.468, *P* < 0.01). LY294002 administration (S&HD&LY) significantly (*P* < 0.01) reversed the infarction to 42.5 ± 2.0%, suggesting a role of the *PI3K*/*Akt* pathway in neuroprotection. A similar pattern of neuroprotection was demonstrated in neurological deficits (Fig. 3). At 24 h of reperfusion, the stroke-only group displayed significant (*P* < 0.05) deficits with 3.9 ± 0.1 in the 5 (Fig. 3a) or 9.7 ± 0.2 in the 12 scoring systems (Fig. 3b). Neurological deficits were significantly reduced by the combination therapy (5 score: 3.0 ± 0.2, *F*_(4,30)_ = 2.944, *P* < 0.01; 12 score: 8.0 ± 0.2, *F*_(4,30)_ = 10.773, *P* < 0.01), but with no improvement with either approach alone. *PI3K*/*Akt* inhibitor reversed the neurological improvement (5 score: 3.6 ± 0.2, *P* < 0.05; 12 score: 8.7 ± 0.2, *P* < 0.05).

### Apoptotic cell death

**Figure 4:**
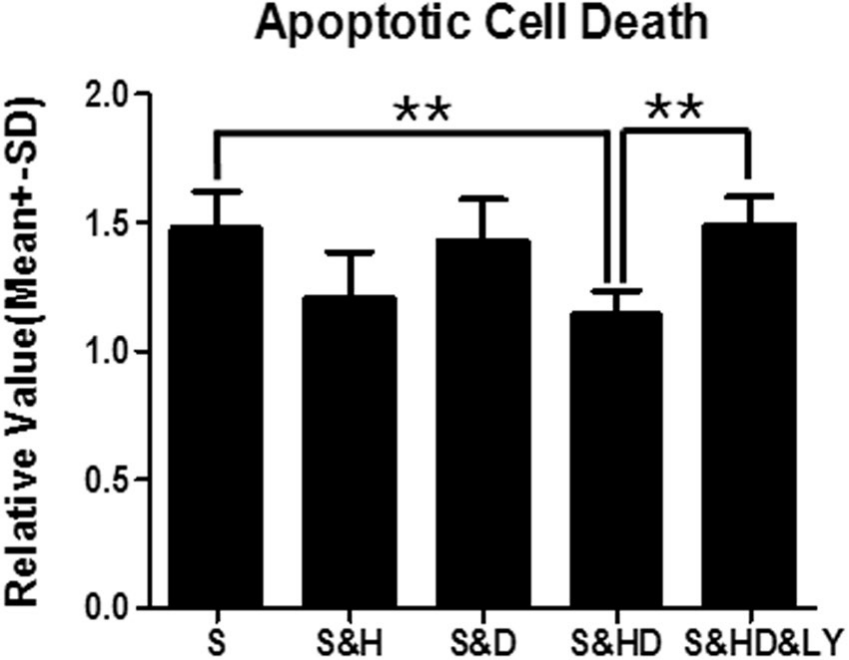
Alleviation of stroke-induced apoptosis (n = 6 per group) with mild hypothermia and C + P combination therapy. ANOVA analyses indicated that combination therapy (S&HD) significantly (**P < 0.01) reduced apoptotic cell death. Monotherapy with mild hypothermia (S&H) only displayed a slight reduction in apoptotic death (*P < 0.05), while C + P monotherapy (S&D) did not significantly increase cell survival. Treatment with p-Akt inhibitor (LY294002) (S&HD&LY) reversed the combination therapy’s effect in neurological improvement (**P < 0.01).

Stroke (S) notably induced apoptotic cell death as compared to the sham group, referenced as 1 (*P* < 0.01) (Fig. 4). Mild hypothermia (S&H) only showed a slight reduction in apoptotic cell death (*P* < 0.05). While monotherapy of phenothiazine drugs (S&D) did not show remarkable change at normal body temperatures, their combination with mild hypothermia (S&HD) significantly reduced cell death (*F*_(4,30)_ = 5.482, *P* < 0.01). This reduction was significantly (*P* < 0.01) reversed by the *PI3K*/*Akt* inhibitor (S&HD&LY).

### Pro-apoptotic protein expression

**Figure 5:**
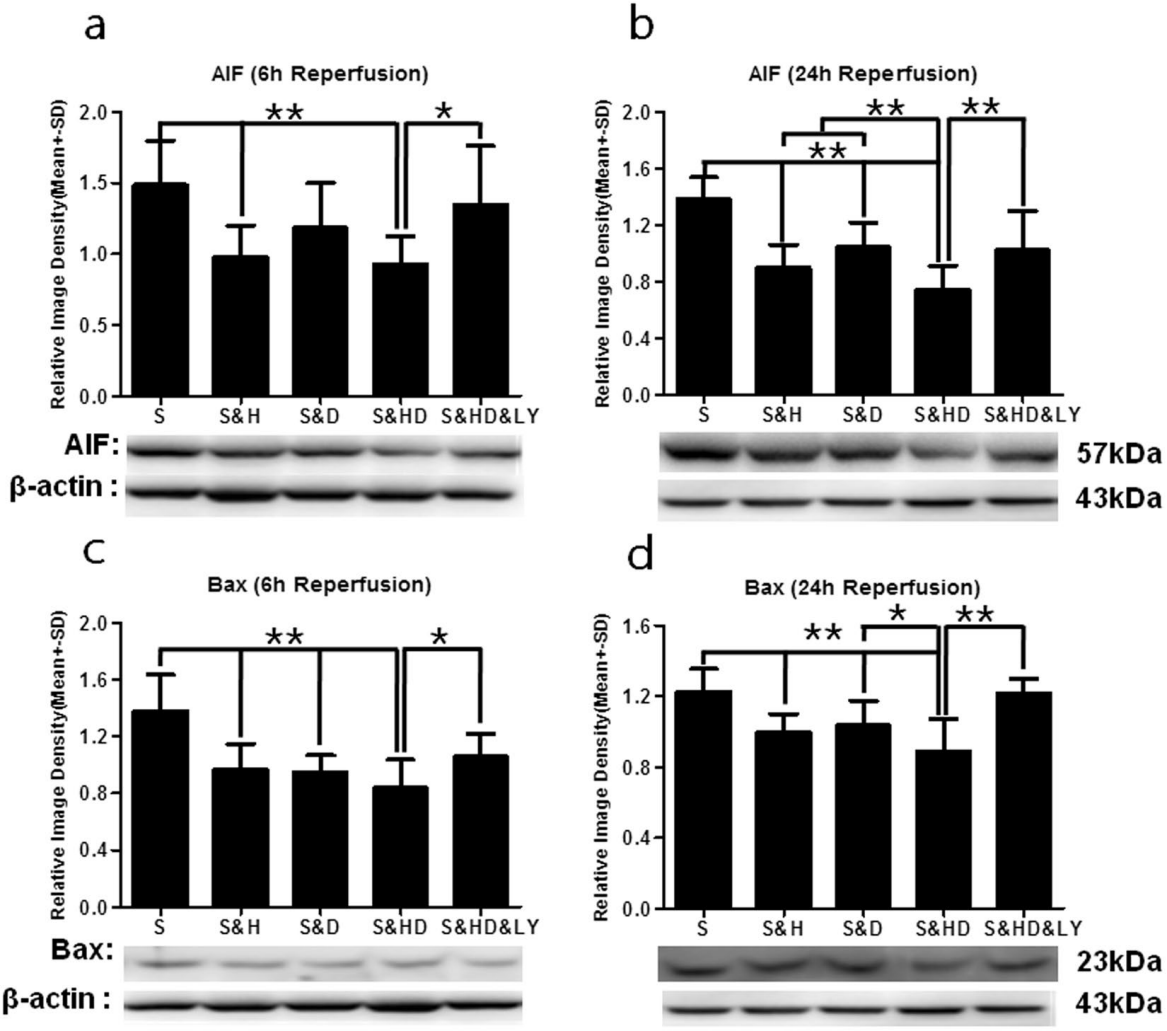
Reduction in pro-apoptotic protein expression with mild hypothermia and C + P combination therapy. ANOVA analyses indicated that there was a significant increase (**P < 0.01) in AIF levels after ischemic stroke (S) at both (**a**) 6 h (n = 6 per group) and (**b**) 24 h (n = 6 per group) of reperfusion. Ischemia-induced increase in AIF levels was significantly reversed (**P < 0.01) with combination therapy (S&HD) at both 6 h (*F*_(4,25)_ = 3.683) and 24 h (*F*_(4,25)_ = 12.009) of reperfusion. Monotherapy with either mild hypothermia (S&H) (**P < 0.01 at 6 h and 24 h) or C + P alone (S&D) (**P < 0.01 at 24 h) led to varying degrees of reduction in AIF expression. *PI3K*/*Akt* inhibitor (S&HD&LY) reversed the combination therapy-induced reduction in AIF levels at 6 h (*P < 0.05) and 24 h (**P < 0.01). Similarly, there was a significant increase (**P < 0.01) in Bax levels after ischemic stroke (S) at both **(c)** 6 h (n = 6 per group) and (**d**) 24 h (n = 6 per group) of reperfusion. Although the expression of Bax was decreased by monotherapies (S&H and S&D) at 6 h (*F*_(4,25)_ = 7.165, **P < 0.01) and 24 h (*F*_(4,25)_ = 8.510, **P < 0.01), combination therapy (S&HD) further enhanced the decrease in Bax expression. *PI3K*/*Akt* inhibitor (S&HD&LY) reversed the combination therapy-induced reduction in Bax levels (*P < 0.05 at 6 h; **P < 0.01 at 24 h). Representative immunoblots are presented.

AIF expression after stroke (S) was significantly increased as compared to sham group, which was referenced as 1, at 6 h (*P* < 0.01, Fig. 5a) and 24 h(*P* < 0.01, Fig. 5b) after reperfusion. Monotherapies with either mild hypothermia (S&H) (*P* < 0.01 at 6 h and 24 h) or phenothiazine drugs alone (S&H) (*P* > 0.05 at 6 h, but *P* < 0.01 at 24 h) led to varying degrees of reduction in AIF expression. On the other hand, their combination (S&HD) showed remarkably decreased AIF expression at both time points (*F*_(4,25)_ = 3.683, *P* < 0.01 at 6 h; *F*_(4,25)_ = 12.009, *P* < 0.01 at 24 h). *PI3K*/*Akt* inhibitor (S&HD&HY) reversed this reduction in AIF levels at 6 h (*P* < 0.05) and 24 h (*P* < 0.01). Similarly, Bax expression after stroke (S) was significantly increased after reperfusion at 6 h (*P* < 0.01, Fig. 5c) and 24 h (*P* < 0.01, Fig. 5d). Although the expression of Bax was decreased by monotherapies (S&H and S&D) at 6 h (*F*_(4,25)_ = 7.165, *P* < 0.01) and 24 h (*F*_(4,25)_ = 8.510, *P* < 0.01), combination therapy (S&HD) further enhanced their neuroprotective properties. As expected, *PI3K*/*Akt* inhibitor (S&HD&LY) reversed the reduction in Bax levels (*P* < 0.05 at 6 h; *P* < 0.01 at 24 h).

### Cleaved Caspase-3 expression

**Figure 6:**
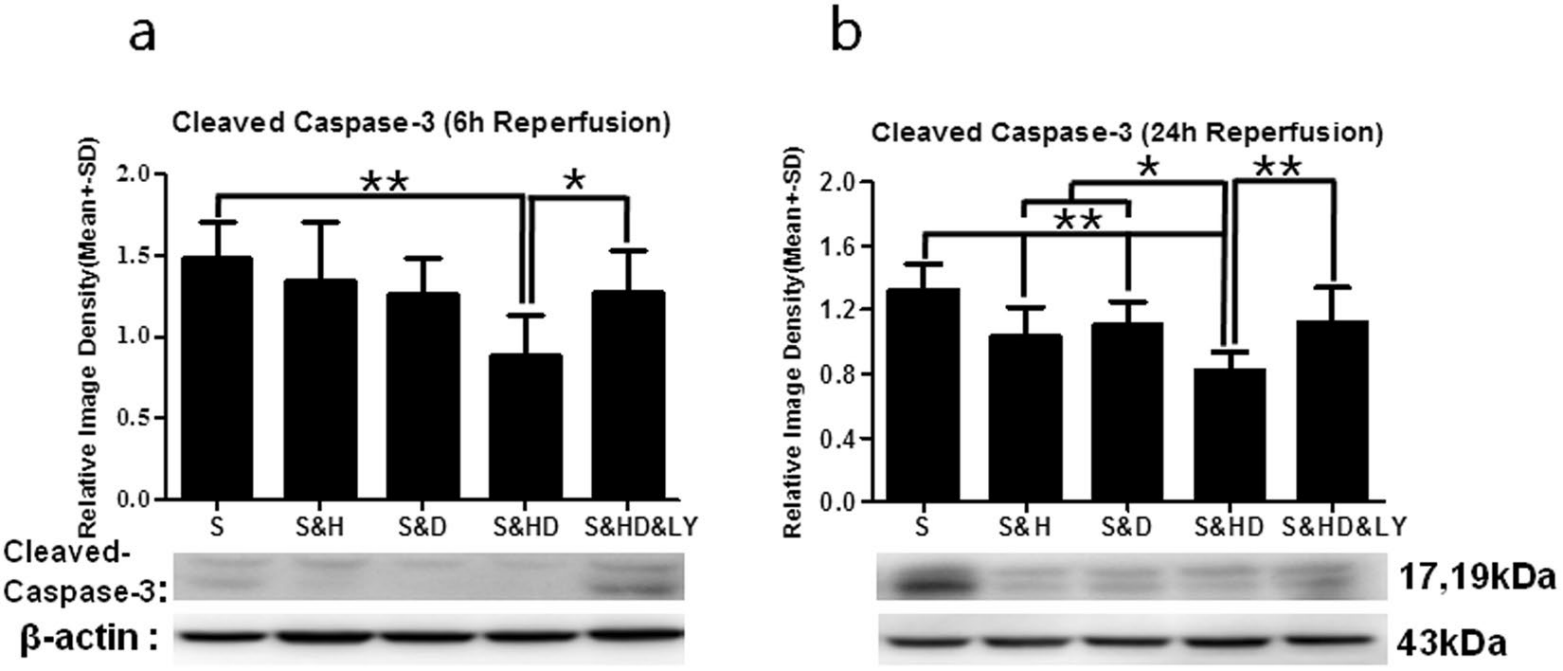
Reduction in cleaved Caspase-3 levels with mild hypothermia and C + P combination therapy. There was a significant increase (**P < 0.01) in cleaved Caspase-3 levels after ischemic stroke (S) at both (**a**) 6 h (n = 6 per group) and (**b**) 24 h (n = 6 per group) of reperfusion. Ischemia-induced increase in cleaved Caspase-3 levels was significantly reversed with combination therapy (S&HD) (**P < 0.01) at both 6 h (*F*_(4,25)_ = 3.432) and 24 h (*F*_(4,25)_ = 6.963) of reperfusion. Mild hypothermia (S&H) (*P < 0.05) or C + P (S&D) (*P < 0.05) only induced a small reduction in cleaved Caspase-3 levels at 24 h after reperfusion. *PI3K*/*Akt* inhibitor (S&HD&LY) reversed the combination therapy-induced reduction in cleaved Caspase-3 expression (*P < 0.05 at 6 h;**P < 0.01 at 24 h). Representative immunoblots are presented.

Cleaved caspase-3 is the active form of caspase-3. Ischemia (S) significantly increased cleaved Caspase-3 protein level as compared to sham control, referenced as 1, at 6 h (*P* < 0.01, Fig. 6a) and 24 h (*P* < 0.01, Fig. 6b) after reperfusion. Monotherapies with either mild hypothermia (S&H) (*P* < 0.05) or phenothiazine drugs (S&D) (*P* < 0.05) only induced a small reduction in cleaved Caspase-3 levels at 24 h after reperfusion. Their combination (S&HD), however, resulted in a notable decrease in cleaved Caspase-3 expression at both time points (*F*_(4,25)_ = 3.432, *P* < 0.01at 6 h; *F*_(4,25)_ = 6.963, *P* < 0.01 at 24 h). *PI3K*/*Akt* inhibitor (S&HD&LY) reversed the reduction in cleaved Caspase-3 expression (*P* < 0.05 at 6 h; *P* < 0.01 at 24 h).

### Anti-apoptotic protein expression

**Figure 7:**
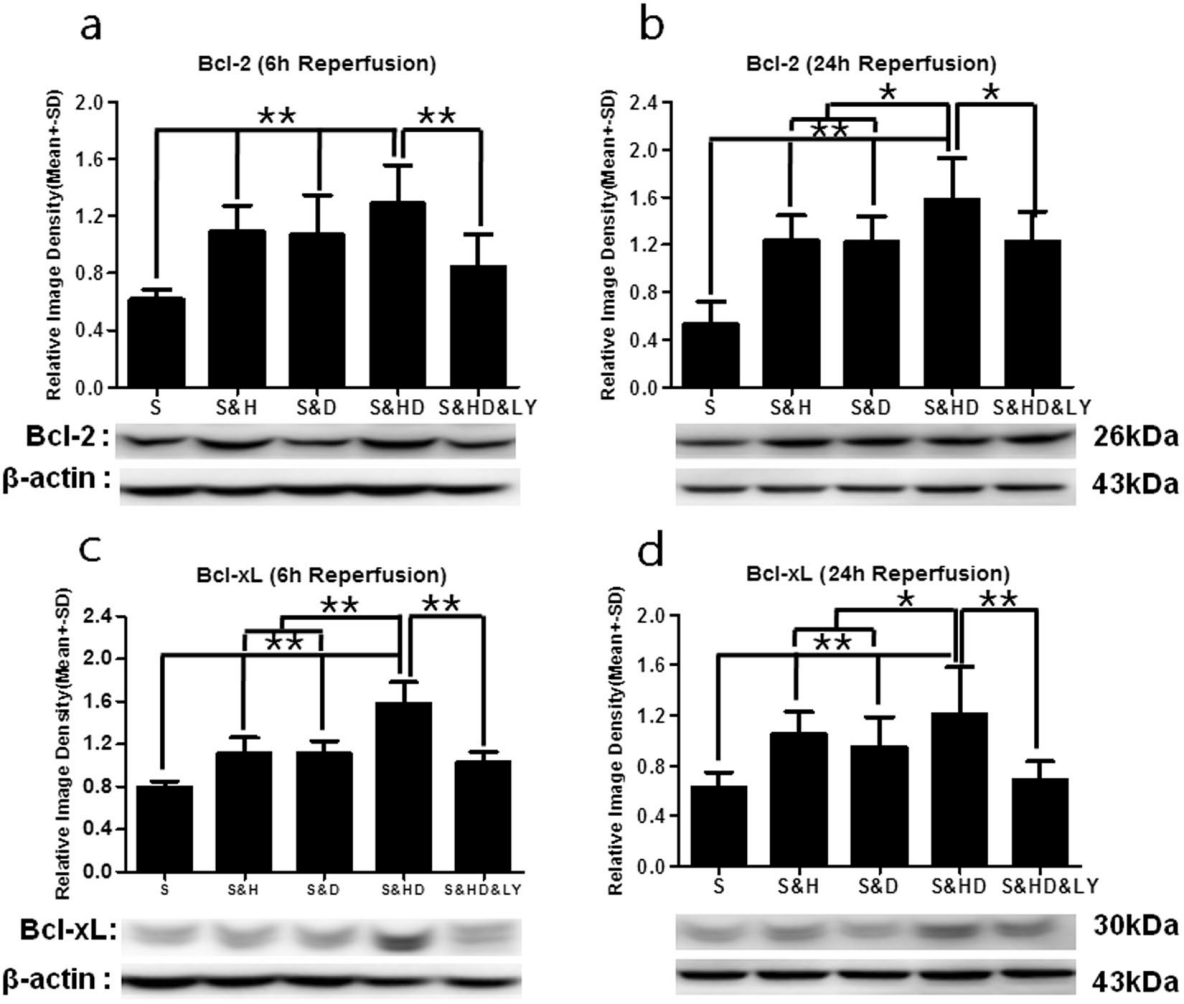
Increase in anti-apoptotic protein expression with mild hypothermia and C + P combination therapy. A significant decrease (*P < 0.05) in Bcl-2 levels was seen after ischemic stroke (S) at both (**a**) 6 h (n = 6 per group) and (**b**) 24 h (n = 6 per group) of reperfusion. Ischemia-induced reduction in Bcl-2 levels was significantly reversed (**P < 0.01) with combination therapy (S&HD) at both 6 h (*F*_(4,25)_ = 8.216) and 24 h (*F*_(4,25)_ = 11.806) of reperfusion. Again, mild hypothermia (S&H) or C + P (S&D) alone only led to a small increase in Bcl-2 expression (**P < 0.01). *PI3K*/*Akt* inhibitor (S&HD&LY) reversed the combination therapy-induced increase in Bcl-2 levels at 6 h (**P < 0.01) and 24 h (*P < 0.05). Similarly, there was a significant decrease in Bcl-xL levels after ischemic stroke (S) at both (**c**) 6 h (n = 6 per group) (**P < 0.01) and (**d**) 24 h (n = 6 per group) (*P < 0.05) of reperfusion. Although the expression of Bcl-xL was induced by either mild hypothermia (S&H) or C + P (S&D) at both time points (**P < 0.01), combination therapy (S&HD) further enhanced Bcl-xL expression (*F*_(4,25)_ = 22.272, **P < 0.01 at 6 h; *F*_(4,25)_ = 6.728, **P < 0.01 at 24 h). *PI3K*/*Akt* inhibitor (S&HD&LY) reversed the combination therapy-induced increase in Bcl-xL levels (**P < 0.01). Representative immunoblots are presented.

Bcl-2 expression was decreased at 6 h (*P* < 0.05, Fig. 7a) and 24 h (*P* < 0.05, Fig. 7b) in the stroke group (S) as compared to sham, referenced as 1. Small increases in Bcl-2 expression by either mild hypothermia (S&H) (*P* < 0.01) or phenothiazine drugs (S&D) (*P* < 0.01) was significantly enhanced by their combination (S&HD) at both time points (*F*_(4,25)_ = 8.216, *P* < 0.01 at 6 h; *F*_(4,25)_ = 11.806, *P* < 0.01 at 24 h). This elevation was inhibited by LY294002 (S&HD&LY) (*P* < 0.01 at 6 h; *P* < 0.05 at 24 h). Bcl-xL expression after reperfusion was also reduced at both 6 h (*P* < 0.01, Fig. 7c) and 24 h (*P* < 0.05, Fig. 7d) in the stroke group (S) as compared to sham (referenced as 1). A small elevation of Bcl-xL expression was induced by either mild hypothermia (S&H) (*P* < 0.01 at 6 and 24 h) or phenothiazine drugs (S&D) (*P* < 0.01 at 6 and 24 h), while combination therapy (S&HD), again, demonstrated a notable increase in Bcl-xL expression at both time points (*F*_(4,25)_ = 22.272, *P* < 0.01 at 6 h; *F*_(4,25)_ = 6.728, *P* < 0.01 at 24 h). Again, this elevation was largely suppressed by the *PI3K*/*Akt* inhibitor (S&HD&LY) (*P* < 0.01).

### P-Akt expression

**Figure 8:**
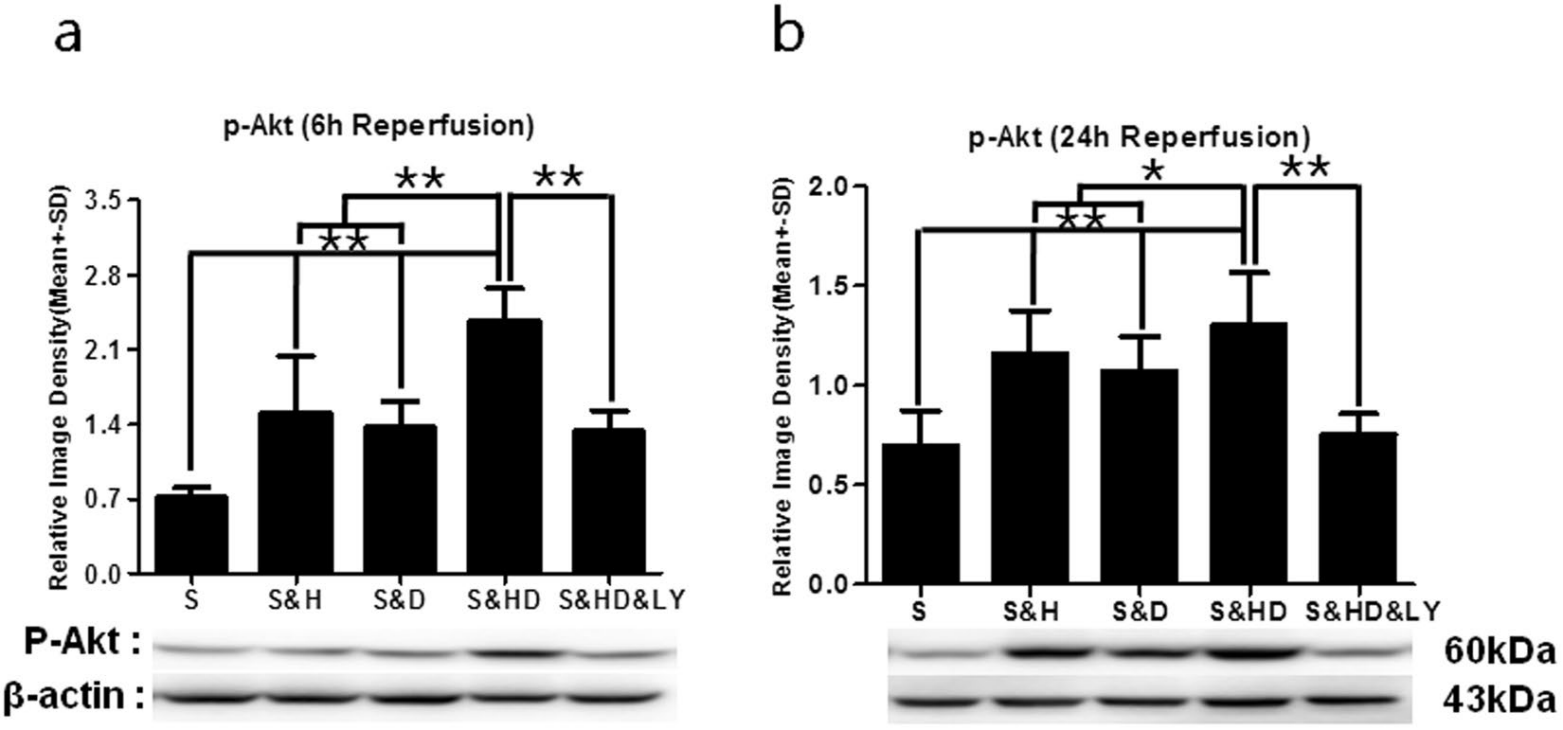
Increase in p-Akt levels with mild hypothermia and C + P combination therapy. There was a significant decrease (**P < 0.01) in p-Akt levels after ischemic stroke (S) at both (**a**) 6 h (n = 6 per group) and (**b**) 24 h (n = 6 per group) of reperfusion. Ischemia-induced reduction in p-Akt levels was significantly reversed with combination therapy (S&HD) (**P < 0.01) at both time points (*F*_(4,25)_ = 22.032 at 6 h; *F*_(4,25)_ = 8.639 at 24 h). Monotherapy with either mild hypothermia (S&H) (**P < 0.01) or C + P (S&D) (**P < 0.01) alone only induced a small increase in p-Akt levels at 24 h after reperfusion. *PI3K*/*Akt* inhibitor (S&HD&LY) reversed the combination therapy-induced increase in p-Akt expression (**P < 0.01). Representative immunoblots are presented.

Phosphorylated-Akt protein expression was significantly decreased at 6 h (Fig. 8a) and 24 h (Fig. 8b) after reperfusion (S) as compared to the sham group, referenced as 1 (*P* < 0.01 at 6 h; *P* < 0.01 at 24 h). Although mild hypothermia (S&H) (*P* < 0.01) or a low dose of chlorpromazine and promethazine alone (S&D) (*P* < 0.01) elevated p-Akt expression at both 6 and 24 h after reperfusion, the elevation of p-Akt expression was largely and significantly enhanced by their combination (S&HD) (*P* < 0.01) at 6 h (*F*_(4,25)_ = 22.032) and 24 h (*F*_(4,25)_ = 8.639). As expected, the *PI3K*/*Akt* inhibitor (S&HD&LY) largely reduced p-Akt levels (*P* < 0.01).

## Discussion

The present study revealed that, while mild and clinically practical hypothermia or low doses of phenothiazine drugs alone did not elicit significant neuroprotection, their combination effectively enhanced neuroprotection. Our therapy had favorable outcomes as illustrated by notable reductions in infarct volume and neurological deficits. The combination therapy may create a pro-survival cellular environment by raising anti-apoptotic (Bcl-2 and Bcl-xL) protein expression while simultaneously reducing pro-apoptotic (AIF and Bax) protein expression. Finally, we demonstrated for the first time that the molecular mechanism behind this neuroprotection could at least partially involve the *PI3K*/*Akt* signaling pathway.

TH with relatively low temperatures at 30–33 °C has been shown to have robust neuroprotective effects following cerebral ischemia^26^. The most common and classical method of hypothermia induction is systemic cooling, including whole-body surface cooling and endovascular cooling^27^, with surface cooling being relatively simpler, less invasive, and more cost effective. However, surface cooling has been limited in clinical practice due to the slow onset of hypothermia, poor control of core temperatures and several side effects^28^. These led to an increasing interest in studying mild hypothermia (~34 °C) to widen its practicality. Unfortunately, it has been shown that using mild temperatures alone confer none to very attenuated neuroprotection^29^. This was further proven in the present study when mild temperature of 33–35 °C was used following 2 h MCAO. For this reason, developing novel approaches to obtain mild and practical TH that can be maintained is warranted.

Hibernation is a phenomenon that is characterized by significant reduction in body temperature and metabolic rate combined with a decrease in cerebral blood flow. This process leads to several favorable physiological changes, including hypothermia, antioxidant defense and hypocoagulation that are beneficial following ischemia^11, 15, 30^. Zhou *et al*. further explained this phenomenon by correlating artificial hibernation with fewer cell death and phagocytosis after traumatic brain injury^31^. Hibernation-like effects could be obtained pharmacologically, and chlorpromazine and promethazine (C + P) are the classical phenothiazine neuroleptics that exert such effects^13, 32^. Recently, we demonstrated neuroprotection induced by C + P in severe transient and permanent ischemic stroke^24^. In this study, C + P treatments conferred neuroprotection by suppressing the damaging cascade of metabolic events, most likely independent of drug-induced hypothermia. We previously also showed a synergetic neuroprotective effect from combining mild hypothermia with low doses of C + P (1.0 mg/kg each)^25^, which was consistent with the finding in the present study. The use of C + P shortened the duration needed to reach the target hypothermic temperature of 35 °C by 20 minutes. This reduced initiation time for hypothermia is very important, especially in the early stage of ischemia, in reducing ischemia/reperfusion injury. However, the low doses of C + P alone did not reduce the infarct volume or neurological deficits.

Apoptosis is one of the many detrimental consequences of cerebral ischemia leading to significant neuronal degeneration. It is triggered by both caspase-mediated and caspase-independent signaling pathways. Members of the Bcl-2 family have been implicated in the process of apoptosis^33^. The intrinsic pathway (also called the Bcl-2 regulated pathway) is stimulated by Bax translocation from the cytosol to the mitochondrial membrane and competition with the Bcl-2 family. Elevation of the ratio in pro-apoptotic factors/anti-apoptotic factors (Bax/Bcl-2) leads to the disruption of mitochondrial membrane and release of cytochrome c. These factors contribute to the activation of Caspase-9, which induces the downstream activation of Caspase-3 and ultimately results in cell death^34–36^. Pucha *et al*. have shown that Bcl-xL prevents the translocation of Bax to mitochondria, thereby inhibiting cell apoptosis^22^. In addition to the caspase pathway, AIF is released from the mitochondria following cell death; a process that can be prevented by the increase in Bcl-2 protein expression^37^. Using our combination therapy, the alterations of pro- and anti-apoptotic proteins, including AIF, Bax, Bcl-2 and Bcl-xL were positively regulated to reduce apoptosis, suggesting that the induced neuroprotection by combination therapy in this study is conferred, at least in part, by modulation of the apoptotic death pathways.

The *PI3K*/*Akt* signaling pathway plays a major role in cellular survival and death^38^. More importantly to our study, Akt has an anti-apoptotic function, especially after stroke. It prevents cell death through its effect on the Bad pathway and inhibits the release of cytochrome *c* from the mitochondria. In detail, Akt phosphorylates Bad and subsequently prevents it from binding to Bcl-xL, thereby preventing apoptosis. Akt has been shown to also modulate apoptosis in other ways, such as directly inhibiting caspase proteases, inhibiting apoptosis signal-regulating kinase 1 (Ask-1), or through the promotion of survival factors^39^. Tsuruta *et al*. have demonstrated that activation of the *PI3K*/*Akt* pathway limits the pro-apoptotic function of Bax and inhibits cytochrome c release from the mitochondria^40^. This study revealed that the administration of the p-Akt inhibitor (LY294002) reversed our treatment’s effects on the apoptotic cellular profile. The p-Akt inhibitor inverted the ratio of pro-apoptotic proteins/anti-apoptotic proteins and enhanced cell death. Although the exact mechanisms of Akt’s inhibition on apoptosis is complex and needs to be further elucidated, our results show that Akt levels decrease during the post-ischemic period, while treatments increased Akt levels significantly. Such finding suggests that the neuroprotective effects of the combination treatment were at least partially regulated through the *PI3K*/*Akt* pathway.

Despite our extensive knowledge of the hallmarks in brain ischemia, we are still in need for protective therapies that can be applied clinically. TH is one of the most studied neuroprotective approaches that have been shown to exert promising clinical outcomes. However, this approach is still far from reaching its full clinical capacity because of its various side effects and limitations. By combining mild hypothermia with phenothiazine neuroleptics, we aimed to bypass the different limitations that have historically narrowed TH’s clinical applications as well as reducing side effects induced by drug monotherapies. We showed that the administration of C + P with mild hypothermia remarkably enhanced the role of TH in the treatment of brain ischemia. Thus, our combination therapy may be a clinically-promising therapy given its efficacy, safety, and ease of use. Our combination therapy has been proven to have neuroprotective effects on brain cells after stroke by creating a pro-survival environment in the cell. The pathway that leads to neural cell loss, however, is very complex and treatment effects on its molecular players are not well understood. Although its effects on Akt are revealed in this paper, other regulators and proteins of the pathway are the subject of future investigation in our lab. In the present study, all treatments were initiated immediately at the onset of reperfusion, which was similar to a stroke patient with thrombolysis or embolectomy as a reperfusion treatment. In a clinical scenario, a patient may develop a stroke where no reperfusion takes place (thrombolysis or embolectomy), or therapy is delayed. Therefore, an ischemia without reperfusion and with a longer term duration will be further investigated in our future study.

## Materials and Methods

### Subjects

This study was performed from May 2015 to Aug 2016. A total of 138 adult male Sprague-Dawley rats (300 to 340 g weight, aged 9 to 10 weeks, Vital River Laboratory Animal Co.) were used. All experimental procedures were approved by the Institutional Animal Investigation Committee of Capital Medical University in accordance with the National Institutes of Health (USA) guidelines for care and use of laboratory animals. All animals were housed in the same animal care facility, with 12-hour light/dark cycles, throughout the study. Animals were randomly picked from cages before surgical preparation and divided into 6 groups: (1) sham-operated group, in which the rats underwent all of the operative process except the middle cerebral artery occlusion (MCAO) (n = 6 * 2); (2) stroke group (Group S), in which the rats were subjected to 2 h MCAO followed by 6 (n = 6) or 24 h (n = 13) reperfusion without treatment; (3–6) stroke with different treatments at the onset of 6 h (n = 6 per group) or 24 h (n = 13 per group) reperfusion: ischemic rats which received only mild hypothermia (Group S&H), intravenous (IV) injection of chlorpromazine and promethazine (Group S&D), combination of mild hypothermia and drug group (Group S&HD), and the *PI3K*/*Akt* inhibitor (LY294002) before combination therapy (Group S&HD&LY). All procedures and data analysis were performed in a blinded and randomized manner. The detail process was as follows: one experimenter produced the MCAO models and neurological scoring under the premise of not being aware of which rats were divided into which group. Another experimenter gave the treatments to the animals. The third experimenter performed the Western blot and enzyme-linked immuno sorbent assay (ELISA). All experimenters were blinded to the designing scheme of the present study. For data analyses, the following animals were excluded: 1) died before end points; 2) skull base hemorrhage; 3) no signs of injury determined by 2,3,5-triphenyltetrazolium chloride (TTC) staining and neurological deficits. In this study, as the first step, we chose a 24 hour time point for outcome assessment after MCAO because these time points were used in previous studies conducted by us and others, which allows a better comparison for the therapeutic effect of combination treatment. Furthermore, the average time of progression from stroke onset has been shown to be between 22 and 48 hours^41^. Thus, both 6 and 24 h was chosen for the present study to investigate molecular mechanisms underlying phenothiazine/hypothermia-induced neuroprotection.

### Focal cerebral ischemia

Anesthesia was induced through a mask with 3% enflurane and maintained with 1–2% enflurane in 70% nitrous oxide and 30% oxygen. Right MCAO was produced using an intraluminal filament^42^. Reperfusion was achieved by the withdrawal of the filament at 2 h of MCAO. The rats were intubated and mechanically ventilated (Rodent Ventilator Model, Harvard Apparatus Inc., Holliston, MA, USA) during the reperfusion and treatment process. Blood pCO_2_, pO_2_, mean arterial pressure (MAP), and blood glucose were monitored throughout the procedure. Heating lamps and pads were utilized to maintain rectal temperature.

### Mild hypothermia

Hypothermia was induced by spraying 75% alcohol onto the rat’s entire body as described previously by us^43^. It is very unlikely that animals ingested any significant amounts of alcohol given the circumstances of our experiment. Based on previous study^44^, contact with exposed skin (as opposed to fur) is minimal, and there is no evidence of clinically relevant transdermal absorption. Hypothermia was administered at the onset of reperfusion. Alcohol spraying was used until rectal temperature dropped to 35 °C. Rectal temperature spontaneously reduced to 33 °C following which a circulating heating pad was added, which then resulted in a gradual rise in rectal temperature. Mild hypothermia with rectal temperatures ranging between 33–35 °C was maintained for 1 h.

### Chlorpromazine and Promethazine Administration

In all ischemic rats, the combination of chlorpromazine and promethazine (1:1) were diluted in 0.9% saline (0.25 mg/ml,1.0 mg/kg each) and injected intravenously at the onset of reperfusion, as described previously by us^25^. To avoid a confounding decrease in temperature, rats were kept in a warm box to maintain rectal temperature at normal range (37.8–38.3 °C). It is important to note that our effort in this study was to detect whether the combination therapy have synergetic neuroprotective effects or the phenothiazine neuroleptics just play a role in promoting hypothermia induction. Thus, we maintained the body temperature at the normal level (about 38 °C) by a regular method which is widely used in other studies as a control. Rats in Group S&HD received both mild hypothermia and the drugs chlorpromazine and promethazine (1:1), which were also initiated immediately at the onset of reperfusion.

### Brain and body temperature monitoring

Brain temperature in the ipsilateral MCA territory was monitored before, during, and after hypothermia by needle thermistor probes (Harvard Apparatus Inc.), which were placed into the cortex and striatum through holes: one made 3 mm lateral and 3 mm posterior to the bregma, and one made 3mm lateral to the bregma on the ipsilateral side, respectively. Rectal and brain temperatures were measured every 10 min until rectal temperature returned to above 36 °C. In addition to the cooling process, rats were placed on a feedback-regulated heating pad throughout the surgical procedure.

### Akt inhibition

To determine a potential role of Akt in TH-induced neuroprotection, we used the PI3K inhibitor-LY294002 ([2-(4-morpholinyl)-8-phenyl-1(4H)-benzopyran-4-one], Cell Signaling Technology, no.9901). This was previously shown to inhibit Akt phosphorylation in many studies and used by us^45^. A microinjection, at a dose of 30 μM (10 μL), was performed into the lateral ventricle ipsilateral to the ischemia over 10 minutes (from bregma: posterior, −1.0 mm; lateral, −1.5 mm; ventral, −4.0 mm), 0.5 h prior to MCAO.

### Cerebral infarct volume

Infarct volume (n = 7 per group) was evaluated at 24 h of reperfusion in all ischemic rats as previously described by us^46, 47^. Six coronal brain slices with a 2 mm thickness were cut for treatment with TTC at 37 °C for 20 min. They were then fixed in 10% formalin solution. In order to minimize errors due to edema, cerebral infarct volume was calculated relative to non-infarcted hemisphere^46, 47^.

### Neurological Deficits

Neurological deficits were evaluated via a modified 5-point scoring system, and sensorimotor integration of forelimbs using the 12-point scoring scale before surgery at 2 h after MCAO as well as 24 h after reperfusion^45^.

### Cell death detection

Apoptotic cell death (n = 6 per group) was measured by quantifying the amount of cytoplasmic histone-associated DNA fragments, using a photometric enzyme immunoassay (Cell Death Detection ELISA; Roche Diagnostics, Indianapolis, IN, USA), as described previously by us^46, 48^. We detected the absorbance of light at 405 nm using a multimode detector to determine the degree of apoptosis in the respective samples.

### Protein expression

Western blot analysis was used to detect expression of pro-apoptotic (AIF, Bax) and anti-apoptotic (Bcl-2, Bcl-xL) proteins as well as p-Akt and cleaved Caspase-3. Tissue samples from the ischemic cerebral hemispheres of all experimental and control groups were harvested at 6 (n = 6 per group) or 24 h (n = 6 per group) after reperfusion, as described previously by us^45^. PVDF membranes after protein transfer were incubated with primary antibodies including rabbit polyclonal anti-Phospho-Akt antibody (1:1000, Cell Signaling Technology), rabbit polyclonal anti-Bcl-2 antibody (1:200, Santa Cruz), mouse monoclonal anti-Bcl-xL antibody (1:200, Santa Cruz), mouse monoclonal anti-AIF antibody (1:4000, Santa Cruz), rabbit polyclonal anti-Bax antibody (1:500, Santa Cruz), and rabbit polyclonal anti-cleaved Caspase-3 antibody (1:1000, Cell Signaling Technology) at 4 °C for 24 h. Membranes were then incubated with the relevant secondary antibody (goat anti-mouse IgG, ZhongShanJinQiao; goat anti-rabbit IgG, ZhongShanJinQiao) for 1 h at room temperature. Equal protein loading was adjusted using β-actin (mouse polyclonal anti-β-actin antibody, Santa Cruz). An ECL system was used to detect immunoreactive bands by luminescence. Quantification of relative target protein expression was obtained using an image analysis program (Image J 1.48, National Institutes of Health, USA).

### Statistical Analysis

Given the large mean difference and small SD in the previous studies, we predicted the effect size for the present study was about 1.00 or above, which suggests a small sample size (n = 6) for the study. All values are expressed as Means ± SD. Statistical analyses were performed using SPSS for Windows, version 16.0(SPSS, Inc.). The differences between groups were assessed using one-way analysis of variance (ANOVA) or Student’s *t-*test with a significance level set at *P* < 0.05. Post-hoc comparison between groups was conducted using the least significant difference (LSD) method.

## Electronic supplementary material

Dataset 1
