# Supplementary material for: Phenothiazines Enhance Mild Hypothermia-induced Neuroprotection via PI3K/Akt Regulation in Experimental Stroke
Source: Sci Rep. 2017 Aug 7;7:7469. doi: 10.1038/s41598-017-06752-5 (PMC5547051; doi:10.1038/s41598-017-06752-5)
Supplement: Supplementary file 1 — Dataset 1 [file 41598_2017_6752_MOESM1_ESM.doc]

**Phenothiazines Enhance Mild Hypothermia-induced Neuroprotection via PI3K/Akt Regulation**

**in Experimental Stroke**

Hong An^1^, Yunxia Duan^1^, Di Wu^1^, James Yip^2^, Omar Elmadhoun^2^, Joshua C. Wright^2^, Wenjuan Shi^3^, Kaiyin Liu^2^, Xiaoduo He^1^,

Jingfei Shi^1^, Fang Jiang^1^, Xunming Ji^1^*, Yuchuan Ding^1,2^

1. China-America Institute of Neuroscience, Xuanwu Hospital, Capital Medical University, Beijing ,China

2. Department of Neurosurgery, Wayne State University School of Medicine, Detroit, MI, USA

3.Cerebrovascular Diseases Research Institute, Xuanwu hospital, Capital Medical University, Beijing, China

***Corresponding Author:**

Xunming Ji, MD, PhD

Department of Neurosurgery,

China-America Institute of Neuroscience,

Center of Stroke, Beijing Institute for Brain Disorders Xuanwu Hospital, Capital Medical University

Beijing 100053, China. Email: [jixm@ccmu.edu.cn](mailto:jixm@ccmu.edu.cn)

[Tel:010-8319](tel:010-8319)8952 Fax:010-8315 4745

SD rats

(300 to 340g weight, aged 9 to 10 weeks)

Neurological scoring identified no injury

Experimental group

(Transient right MCAO)

Sham group

Neurological Scoring

2 h

2 h

No treatment

PH

(33-35^0^C for 1 h)

C+P

( 0.25mg/ml,

1.0mg/kg each)

Combination therapy

Combination therapy+p-Akt inhibitor

Neurological Scoring before sacrifice

Expressions of p-Akt, cleaved Caspase-3, pro-apoptotic (AIF, Bax) and anti-apoptotic proteins (Bcl-2, Bcl-xL) evaluated by Western blot at 6 (n=6 per group) and 24 h (n=6 per group) after reperfusion.

Anesthesia

TTC staining at 24 h after reperfusion

(n=7 per group)

Apoptotic cell death detected by ELISA at 24 h after reperfusion

(n=6 per group)

Anesthesia

MCAO: middle cerebral artery occlusion

PH: Physical Hypothermia

C+P: Chlorpromazine and Promethazine

AIF: apoptosis-inducing factor

ELISA: enzyme linked immunosorbent assay

TTC :　2,3,5-triphenyltetrazolium chloride

Exclusion criteria:

1) died before end points;

2) skull base hemorrhage;

3) no signs of injury determined by TTC staining and neurological deficits.
